# Heat Shock Proteins: Dynamic Biomolecules to Counter Plant Biotic and Abiotic Stresses

**DOI:** 10.3390/ijms20215321

**Published:** 2019-10-25

**Authors:** Saeed ul Haq, Abid Khan, Muhammad Ali, Abdul Mateen Khattak, Wen-Xian Gai, Huai-Xia Zhang, Ai-Min Wei, Zhen-Hui Gong

**Affiliations:** 1 College of Horticulture, Northwest A&F University, Yangling 712100, China; Saeed_ulhaq@nwsuaf.edu.cn (S.u.H.); abidagriculturist@gmail.com (A.K.); alinhorti@yahoo.com (M.A.); gaiwenxian@163.com (W.-X.G.); 2016060124@nwsuaf.edu.cn (H.-X.Z.); 2 Department of Horticulture, University of Agriculture Peshawar, Peshawar 25130, Pakistan; Mateen@aup.edu.pk; 3 College of Information and Electrical Engineering, China Agricultural University, Beijing 100083, China; 4 Tianjin Vegetable Research Center, Tianjin 300192, China; waimin163@163.com; 5 State Key Laboratory of Vegetable Germplasm Innovation, Tianjin 300384, China

**Keywords:** chaperone, co-chaperone, heat shock factor, biotic stress, abiotic stress, protein folding, stress resistance

## Abstract

Due to the present scenario of climate change, plants have to evolve strategies to survive and perform under a plethora of biotic and abiotic stresses, which restrict plant productivity. Maintenance of plant protein functional conformation and preventing non-native proteins from aggregation, which leads to metabolic disruption, are of prime importance. Plant heat shock proteins (HSPs), as chaperones, play a pivotal role in conferring biotic and abiotic stress tolerance. Moreover, HSP also enhances membrane stability and detoxifies the reactive oxygen species (ROS) by positively regulating the antioxidant enzymes system. Additionally, it uses ROS as a signal to molecules to induce HSP production. HSP also enhances plant immunity by the accumulation and stability of pathogenesis-related (PR) proteins under various biotic stresses. Thus, to unravel the entire plant defense system, the role of HSPs are discussed with a special focus on plant response to biotic and abiotic stresses, which will be helpful in the development of stress tolerance in plant crops.

## 1. Introduction

Plants are sessile organisms and are subjected to various threats, both biotic and abiotic. These stresses, individually or in combination, result in huge losses in terms of growth, development, and yield and sometimes threaten the survival of the plant [1]. Plants continuously confront harsh environments like high/low temperatures, drought, salts, heavy metals, light, flooding and physical wounding [2,3,4,5]. Biotic stresses like pathogens (Viruses, Bacteria, Fungi) and pests such as nematodes, insects, and rodents also restrict plant productivity [6,7,8,9,10]. Negative effects of these stresses on the plant germination [11], are stunted growth [12,13], sunburn and scorching of leaves [14], loss of photosynthetic pigment, decreased production of photo-assimilates, and depletion of carbohydrate reserves which results in starvation [15,16,17,18]. Abiotic stresses also negatively affect the reproductive characteristics of plants by enhancing male sterility [19] and increasing premature flower and fruit drop [20] which results in significant low yield and quality. It has been reported that the increase in temperature by 1 °C results in a 4–10% yield decrease [21]. As a consequence of these stresses, reactive oxygen species (ROS) are produced which lead to oxidative stress and, ultimately, results in cell death. ROS could be singlet oxygen (^1^O_2_), superoxide radical (O_2_^•−^), hydrogen peroxide (H_2_O_2_) and hydroxyl radical (OH ^−^), which are produced in cell organelles such as mitochondria, peroxisomes and chloroplasts in oxidative stress situations and react with all types of macromolecules like pigments, proteins, lipids and DNA [22,23].

Plants respond morphologically to elevated temperature and light stress by changing the leaf orientation [13], anatomically by altering stomatal conductance and increased leaf pubescence [24,25], and phenologically by shifting and improvising the developmental stages to escape the abiotic stress condition [26]. Plants also change the metabolic processes and physiology to retain root hydraulic conductance [27], accumulation of the compatible osmolytes, such as sugars, sugar alcohols, proline and phenolic compounds under saline and water-logged conditions, as well as high temperature and water deficit conditions [28]. Moreover, plants manage to maintain photosynthetic machinery [29] by changing their assimilate partitioning a shift occurs from symplastic to apo-plastic [30]. During the onset of the stress situations, plants also improvise the hormonal balance of abscisic acid (ABA), ethylene, and salicylic acid (SA) as a signaling molecule in the systemic acquired resistance. Similarly, jasmonic acid (JA) and other steroids enhance stress tolerance and resistance [31]. Furthermore, secondary metabolites, such as isopropanioid, carotenoid, flavonoid, anthocyanin, lignin, and isoprenoids [30,32], also are produced and accumulated.

Besides these adaptations, plants also have sophisticated adaptive systems at the cellular and molecular levels. During the onset of stress, plants reduce the synthesis of normal protein production, and transcribe and translate heat shock proteins (HSPs). Added to transcriptional regulations, plants also have some sophisticated post-transcriptional modifications which help the plant to cope with these stresses, such as alternative splicing and micro RNA (miRNA). Alternative splicing, which generates multiple copies from a single gene, helps the plants to mitigate abiotic stresses [33]. One of the important plant post-transcriptional modification strategies is the miRNA, which binds to the mRNA at any point to repress translation or direct cleavage of the mRNA. Some of the miRNAs also are involved with abiotic stress tolerance [34].

HSPs have been identified for a long time in cellular biology as proteins, which concentration dramatically increases when cells are grown at higher temperatures. Now, it is established that these are proteins that help newly synthesized proteins to fold, or to protect proteins that might mis-fold and thereby lose their potential functional conformation during a stress event, such as biotic and/or abiotic stress [35]. These stress-responsive biomolecules act as molecular chaperones which perform under stress situations [36]. Generally, proper protein folding, unfolding and transport, in conjunction with their localization in the cell, and, subsequently, disposal and degradation of the non-native proteins, are the main functions of HSPs [37,38,39]. HSPs are important in the plant life cycle as its role clearly extends beyond the protection from biotic and abiotic stresses. Although HSPs (with the exception of ubiquitin) were first characterized due to their response to high temperatures, now many HSPs are found at normal, non-stressed cells, are produced at particular stages of the cell cycle, or during development in the absence of stress [40]. HSPs beside stress-responsive biomolecules, also are involved in plant growth and development under normal conditions, like the flowers, seeds and fruits set, and the development [41], tuberization [42,43,44,45] and nutrient uptake [46]. HSPs are found in different compartments of the cells, such as cytoplasm, and nucleus, and cell organelles like mitochondria, chloroplasts and endoplasmic reticulum [47,48].

It is evident, now, that HSP expression is controlled by transcription factors known as the heat shock factor (HSF). Seen in plants, among the HSF classes, HSFA positively regulates plant tolerance to anoxia, heat, osmotic and oxidative stresses [49]. Found in tomato plants, HSFA1 is considered a master regulator of signal perception, transduction and controlling the expression of stress-responsive genes, including HSPs [4,50], thus, increased expression of HSPs and other stress responsive genes in conjunction with HSFs play an important and significant role in modifying physiological and biochemical processes, which leads to the development of tolerance to stresses [51,52].

Earlier, HSPs were believed to produce under heat stress, as the name indicates, but now it is established that these biomolecules are produced in response to various biotic and abiotic stresses. HSPs show response to biotic and abiotic stress situations by up- or down-regulation, but, sensing signals and transduction, particularly in biotic stress, still needs to be explored [53]. HSPs are grouped into different classes based on their molecular weight in kilo Dalton (kDa), such as HSP100, HSP90, HSP70, HSP60, and small HSPs, respectively [50,51,52,54,55]. These chaperone families are found in a wide variety of organisms and are involved in maintaining cell homeostasis, transportation of newly synthesized proteins across cell organelles, and folding—preventing mis-folded, denatured and aggregated proteins caused by stress conditions [37,56,57]. Industrialization and urbanization have resulted in climate change and global warming, which pose serious challenges for plant growth, development, yield, and quality and sometimes threaten the plants existence. Considering such a scenario, plant biologists and researchers are trying to investigate, identify and confirm certain traits and characteristics that are related to stress tolerance. Similarly, scientists also are employing the recent approaches of omics techniques in the development of transgenic plants through the incorporation and manipulation of the stress-related genes. Regarding this, HSPs are of wide function, both in development as well as in stress tolerance. We focus on, and compile, various scientific developments that took place in the recent past in respect to role of the HSP families in different crop plants against various biotic and abiotic stresses, which will be helpful in future research regarding the development of stress-tolerant varieties.

## 2. Occurrence of HSP in Plants

The heat shock response is not unique in plants. It was first discovered in the early 1960s by an Italian scientist, F. Ritossa, in *Drosophila melanogaster*, while working on high-temperature stress [58,59]. HSPs were studied in *Saccharomyces cerevisiae,* by McAlister et al. (1980) [60], *Escherichia coli,* by Yamamori et al. (1982) [61], and plants (*Glycine max*), Lin et al. (1984) [62]. A comparison of the response in different organisms has shown that HSPs are conserved highly across organisms [63]. The evolutionary conservation of the heat shock response shows that the production of HSPs is a fundamental and essential process in all organisms [51].

**Figure 1:**
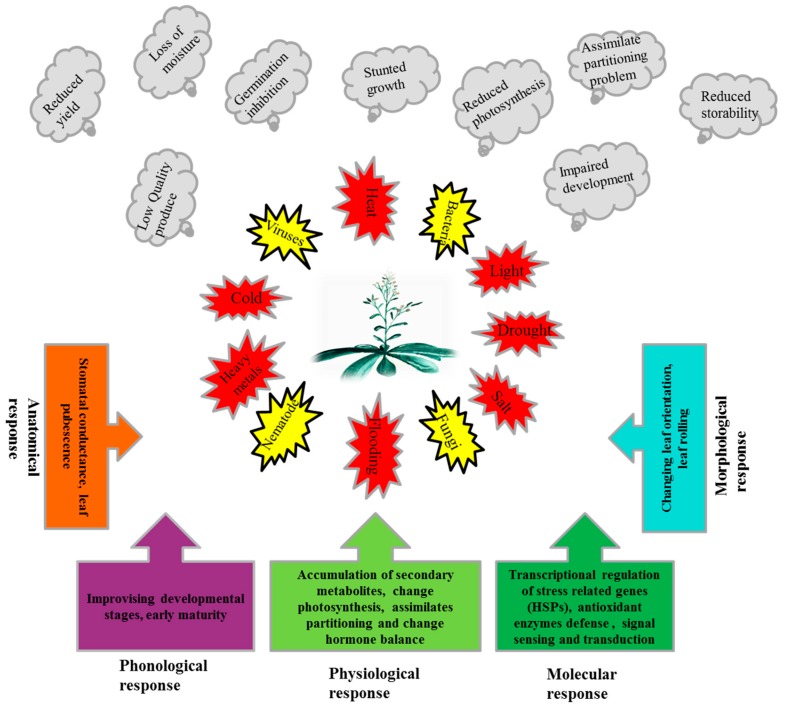
Exposures of plants to different biotic and abiotic stresses, adverse effects of these stresses on plants and response mechanisms of plants to these stresses.

Plants, due to their sessile nature, have evolved different efficient strategies to ensure their generations in the challenging competitive environment. The numbers of stress related genes, including HSPs, are greater in plants than other organisms due to whole genome duplication and gene retention in their evolutionary past [64]. Additionally, plants contain chloroplasts which have their own genome and de novo protein synthesis machinery [65]. A strategy of the plant to cope with stressful situations (Figure 1), is the synthesis of normal protein reduced while the synthesis of stress-related proteins i.e., HSPs, is enhanced.

Many HSPs have been reported in a wide range of organisms from prokaryotes to eukaryotes [63,66]. These HSPs across the organisms are conserved highly, with little difference except for HSP33, which differs in plants from that in bacteria [67]. The genes which encode different HSPs are found in different cell compartments, such as the nucleus, mitochondria, chloroplast, endoplasmic reticulum and cytosol [68]. Similarly, the accumulation of these HSPs in different parts of the cell also depends on the intensity of the stress. Nuclear HSPs, for instance, are accumulating in the cytosol at the lower and higher temperatures of 27 °C and 43 °C, respectively, while the same aggregate in chloroplast is at 37 °C [47]. Different HSPs are found and differentially expressed in different species, even in different genotypes but in the same species, as investigated by Korotaeva et al., (2001) and Nieto-Sotelo et al., (2002) [69,70] in small HSPs where five sHSPs showed response to a higher temperature (42 °C) in maize but only one expressed in wheat and rice. Likewise, HSP68 expressed in mitochondria under stress situations in potatoes, tomatoes, and soybeans [71]. Some HSPs showed a tissue-specific response to stress situations; HSP101 was expressed more in reproductive parts like tassels, ear, and endosperm, than in vegetative parts like leaves and roots in maize [72]. Some HSPs responded differently to the varying length of stresses. As Heckathorn et al., (1989) [73] reported, in HSP45, a nuclear protein accumulated in the chloroplast at a 3 h exposure to heat stress, which returned to its native state after removal of stress. Similarly, HSPs triggered differently with different development stages. HSP45, for example, showed a response in the whole plant to a stress situation, while HSP64 and -72 only showed expression in the reproductive parts i.e., pollens [74,75]. It can be deduced that these are the key regulators which show different responses to varying levels of stress in different parts of the plants.

## 3. Classification and Nomenclature of HSPs

Heat shock proteins are conserved in almost all organisms from bacteria, to fungi, plants and animals, including human beings. HSPs are classified and named based on the molecular weight in kilo Dalton (kDa), which ranges from 8–200 kDa [66]. Based on molecular weight, HSPs generally are classified into the following sub-families: HSP100, HSP90, HSP70, HSP60, and small HSPs [66,67,76,77,78]. The names for HSPs in bacteria are different from plant HSPs, but the classification remains the same on the basis of molecular weight [51] (Table 1). Characteristic features of plant HSPs are nuclear binding domain-I, -II and a middle domain which is preceded by an N- terminal region and followed by a C -terminal extension [79].

## 4. Regulation of HSP

**Figure 2:**
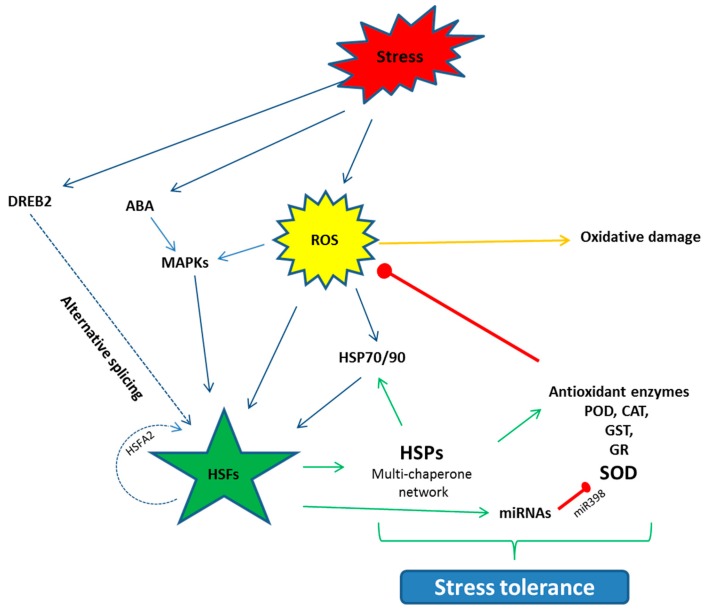
Schematic diagram of the activation of the HSFs and their interaction with the other pathways to counter stress situations. HSFs are activated directly or indirectly through the event of alternative splicing. HSFs further regulate the down-stream HSPs, antioxidant enzyme genes and miRNAs, which help the plants to develop stress tolerance. Arrows denote the positive while red bars stand for negative interaction. ROS (Reactive oxygen species), ABA (Abscisic acid), MAPK (Mitogen-activated Protein Kinase), DREB (Dehydration responsive element binding protein), POD (Peroxidase), CAT (Catalase), GST (Glutathione S transferase), GR (Glutathione reductase), SOD (Superoxide dismutase).

When plants are exposed to stress, the synthesis of normal proteins is decreased while the expression of special genes are up-regulated and, as a result, the synthesis of HSPs is triggered. To respond to stresses, the transcriptional regulation of HSPs is called the heat shock response (HSR) [77]. This HSR is regulated by HSFs in the promoter region, which bind to *cis*-acting elements known as HSE (Heat shock elements) [80,81]. HSFs are classified as three types: HSFA, B, and C; the functions of these classes vary from each other. Among the HSFs, HSFA regulates the HSPs cycle, which is found in the cytosol in a monomeric state. The activity of the HSFA, under normal conditions, is regulated negatively by HSP90s and are checked in the form of phospho-proteins [82]. During the onset of stress, this repression is reversed and HSP90 dissociates and changes into a functional trimer state. This HSFA homo-trimer then binds to the HSE in the promoter region [83], transcription occurs and HSPs are synthesized [84]. Among the HSFA, HSFA1 acts as the master regulator in tomatoes [50]. HSFA2 is structurally and functionally the same as HSFA1, but only is expressed in stressed plants. Under stress situations, HSFA2 makes a super activator hetero-oligomer structure with HSFA1, which is more efficient than the individual HSFs, which not only regulate the down-stream stress related *HSP* genes, but also the protective enzyme genes such as *GST, GR, POX* and *APX* [49,52]. Some studies also report that *HSP* gene expression positively regulates the protective enzyme activities. Seen in *Arabidopsis*, over expression of HSP17.8 enhanced the SOD activity and, in tobacco, HSP16.9 increased the activities of POD, CAT and SOD [85]. Post-transcriptional modification, such as alternative splicing, also regulate the HSFs. HSFA2 under heat stress, for instance, binds to its own promoter region and activates its own transcription in a positive auto-regulatory loop. Similarly, HSFA are regulated by DREB2 under stress, which in turn regulates the stress related genes in many plants [33] (Figure 2). Similarly, miRNAs also play a vital role in the stress response by down-regulation of stress-related genes. Some of the miRNAs are reported to have positive regulation in drought, cold, salinity, hormones and nutrient starvation stresses such as miR159, miR319, miR395, miR402 [34]. Conversely, in *Arabidopsis* short term heat stress, miR398 negatively regulates the expression of CSD1, CSD2 and CCS, which yield the SOD [85].

## 5. Role of HSPs in Plant Defense

Plants are subjected to various biotic and abiotic stresses, singly or in combination, which adversely affect plant growth, development, and survival [86]. To cope with these stresses, plants have evolved various defense strategies i.e., physical [13], anatomical [25], and physiological [27,87]. Plants also respond to stress situations at the molecular level by altering gene expression, synthesis of stress-related biomolecules and proteins including heat stress proteins, to enable plants to ensure their generation in challenging situations.

### 5.1. Biotic Stress Tolerance

Plant growth, development, yield, and quality are affected adversely by several biotic factors such as pathogenic bacteria, fungi, viruses, and nematodes. Biotic factors, directly deprive their host plants of their nutrients, which result in reduced plant vigor, growth, productivity and sometimes leads to death of the host plants. Biotic stresses are major cause of pre- and post-harvest losses. Animals have an immune system, which helps them to adapt to biotic stresses such as new diseases and memorized the past infections, while plants lack such a system. Although plants lack this adaptive immune system, they have evolved several sophisticated strategies to counteract these biotic stresses [88]. These defense mechanisms are stored in the plant’s genome at the genetic level, which encode thousands of stress resistance genes. One of the adaptive systems plants employ in response to biotic stress is through regulation of HSPs (Table 2). HSP response to biotic stresses depend on the nature of the causal organisms and plant genotypes, either susceptible or resistant, and the developmental stage [6].

#### 5.1.1. Fungi

The phyto-pathogenic fungi not only cause devastating epidemics, but also cause serious yield losses which challenge global food security. During invasion, these pathogens employ different enzymes, toxins and secrete effector proteins. Different invading fungi have different types of interaction with the host, it could be biotrophic, necrotrophic or hemi-biotrophic [89]. To respond, plants have evolved different defense strategies, signal perception, transduction and activation of the immune system, including stress-related HSPs.

Tomato 3 species exposed to high and low temperatures along with infection by *Phytophthora infestans*, results in the up-regulation of *HSP70* genes and the increased synthesis of HSP70 proteins. An increase in the temperature increases the mRNA and *HSP70* genes, however, the increase of HSP70 proteins only occurs in susceptible tomato varieties [90]. Plant defense is affected directly by HSP-hijacked transport of the pathogens to the chloroplast where it forms a large structure that weakens the plant defense system, as reported in *Pseudomonas syringae* type III effector Hop II [91]. Small HSPs showed differential responses to infection by pathogenic fungi in rice, which were growth stage-specific [92], in relation to *Magnaporthe grisea* infection. Four of the HSPs (HSP16, 17, 18.1 and 18.2) were up-regulated, while four HSPs (HSP16.6, 17.8, 18.8 and 22) were down-regulated. Knockdown of some of the HSPs has affected the severity of the fungal infection in some cases. Van et al., (2010) [93] studied the *Fusarium oxysporum* infection in relation to HSP20 and some PR proteins. When HSP20 was silenced, the more severe infection was noted, although the PR proteins were there. A similar trend was noted by silencing the HSP17.6 in tomato *Rhizopus nigricans* post-harvest fruit decay [94]. Ahmad et al., (2015) [95] reported powdery mildew infection development with suppression of HSP16.9 in genotype SEP0105. Some researchers have established that HSPs are more effective against the pathogen in a complex as compared to individual HSPs. *Arabidopsis* HSPs along with PR proteins were more effective against *Rhizoctonia solani*. *HSP20* expression along with PR proteins was more than 10 folds in a pathogen resistant genotype AG-8 but was normal in pathogen susceptible genotype AG2-1. Prominent performers out of these *HSP20* genes were *HSP17.4* and -*17.6,* overexpressed and silenced, respectively, have the same response for both genotypes [96,97]. One report showed the possible degrading or knockdown mechanism of HSP by the pathogen while developing an infection in the host, as evident in the proteomic study of apple fungus *Venturia inaequalis,* which displayed knockdown of the *HSP21* during infection [98]. ROS triggered the induction and accumulation of HSP72 and HSP75 in two tomato genotypes against abiotic stresses and fungus *Oidium neolycopersici* [99].

HSPs also are involved in positive interaction with other defense-related proteins to develop resistance against biotic stresses as Yogendra et al., (2015) [100] reported in potato against powdery mildew. *HSP17.8* and *WRKY* simultaneously were regulated transcriptionally in the resistant potato genotypes where HSP chaperone activity kept the defense-related proteins active during abiotic stress. A recent report on powdery mildew infection in sunflowers, susceptible and resistant genotypes in relation to HSP70 demonstrated that HSP70 and other pathogenesis and defense-related proteins were more expressed and accumulated in the resistant genotypes than in the susceptible ones [101].

#### 5.1.2. Bacteria

Plant pathogenic bacteria are important biotic factors that limit plant productivity. Many plant pathogenic bacteria employ a type III secretion system to cause an infection through injection of different effector proteins into host plant cells. Plants respond to many phyto-pathogenic bacteria by pathogen-associated molecular patterns (PAMPs) as a basal defense response [102]. Plants also have evolved effector-triggered immunity (ETI) where resistance (R) protein can recognize the pathogen effector proteins and effective defense is developed against the invading pathogen, particularly the bacteria [6]. HSPs play an important role against virulent bacterial strains, as studied in tobacco against the *Ralstonia solanacearum* infection, where HSP17 was induced and accumulated in the virulent strain as compared to the avirulent strain [103]. The *HSP17* gene, which corresponds to the HSP20 protein, had PR1 and PR4 expression increased soon after infection, even in the avirulent strain of the pathogen. *HSP20* knockdown in the presence of the PR protein resulted in the virulence of non-pathogenic *R. solanacearum*. The same pattern was reported in HSP20′s involvement in resistance against compatible and incompatible *Xanthomonas campestris* in peppers and oranges, which showed HSP20 responded in basal resistance against the biotic stress [104]. Seen in *Arabidopsis*, six of the small HSPs were reported as down-regulated to *Pseudomonas syringae* [105] while, later on, it was explored that this down-regulation was due to salicylic acid, as the pathogen also mediated the SA pathway to cause infection [106]. HSP90, in contrast, has a positive interaction with *Ralstonia solani* in tobacco. HSP90, PAR1, and SGT1 were silenced; only the PAR1 silenced plants showed infection. HSP90- and SGT1-silenced plants resulted in lower infection, although PAR1 accumulation was found in the silenced plants, which showed a positive interaction of HSP90 with the infection [107].

#### 5.1.3. Viruses

Viruses, upon entry into the plant cell, use the plant cell machinery to perpetuate and spread into the neighboring cells and quickly throughout the plant [9]. *Cucumber necrosis virus* (CNV) infection in the *Nicotiana benthamiana* resulted in higher *HSP* transcripts, which resulted in more accumulation of HSPs. This high concentration of HSP70 positively regulated CNV genomics RNA, coat protein and viron accumulation and infection [108]. Likewise, in *Tomato yellow leaf curl virus* (TYLCV), nuclear material was lowered after the knockdown of *HSP70* genes [109]. The expression of HSP is not only specific to pathogen strains but also depends on the time after inoculation.

Occurring in *Arabidopsis*, regarding five viral strains at an interval of 1-day post-inoculation, HSP17.4 and HSP 17.6 were induced in only two strains after one day and showed a delayed response to the other three strains after three days, when studying profile expression by a microarray technique [110]. Similar results also were reported by Senthil et al., (2005) [114], who studied the heterologous microarray of potatoes in response to a virulent strain of *Sonchus Yellow Net Virus* (SYNV) and a virulent strain of *Impatiens Necrotic Spot Virus* (INSV) in model plant tobacco. HSP18 and HSP20 were up-regulated at four days after inoculation, while were lower at the fifth day post-inoculation to INSV; however, they were not induced by the SYNV strain. Viruses in some cases target the subcellular localization and expression pattern of HSP to develop an infection in the host cells. Li et al., (2015) [126] studied HSP20 in relation to *Rice Strip Virus* (RSV) where an invading pathogen infected the HSP20 cellular location with a large virus protein RdPd. This negative interaction possibly was responsible for virus spread and infection in the host cell. To contrast, HSP70 had a positive interaction with RSV and *Tobacco Mosaic Virus* (TMV), where heat treatment also increased the viral infection. HSP70, when silenced, also lowered the viral infection [127]. Some viruses need specific protein in the host cell for assembly and to establish infection in the host cell; for instance, *Red Clover Necrotic Mosaic Virus* (RCNMV) needs 480 KDa functional proteins to replicate in the host cell with the help of HSP70 and HSP90. This protein synthesis was checked by HSP70 knockdown and so was the infection, although a large complex of viral RNA was accumulated in the host cell but was non-functional [113]. A similar pattern was reported by Chen et al., (2014) [112] where HSP70 accumulation was noticed under viral infection and heat stress in tobacco. When cytoplasmic HSP70 was silenced, no viral infection was noticed.

One interesting report pointed out the role of *Tomato Yellow Leaf Curl Virus* (TYLCV) in controlling PCD by deactivation of tomato HSFA2. This inactivation resulted in HSP90 silencing, which mitigated the PCD and kept the plant healthy for its replication and infection at a later stage. Furthermore, the *Root knot nematode* coat protein transport was associated with HSP70 from the cytoplasm into the nucleus of a tomato plant. Silencing of HSP70 in the tomato plant controlled the viral infection. Conversely, HSP90 knockdown promoted viral infection [10,111]. A recent study on *Potato Virus Y* (PVY) in potato thermo-tolerant and sensitive genotypes under heat stress, HSPs was induced in both genotypes. Regarding thermo-tolerant genotypes, the expression of *PR* genes was also high, resulting in less viral infection [117]. Resistance against viral infection in Cytosinpeptidemycin application for the control of *Rice Black Streaked Dwarf Virus* (RBSDV), along with other defense-related genes, antioxidant enzymes and HSP also were up-regulated to control the virus infection [115].

#### 5.1.4. Nematodes

Generally, nematodes species such as the root-knot nematode (*Meloidogyne spp.*), root-lesion nematode (*Pratylenchus spp.*) and cyst nematodes (*Heterodera and Globodera spp.*) are more detrimental to agricultural crops [8]. HSPs are involved in resistance to phyto-nematodes. Li et al., (2015) [7] analyzed RNA-Seq data of *Gossypium hirsutum* after *Rotylenchulus reniformis* infection at a three day interval up to 12 days. Twenty-three HSPs were induced in susceptible genotypes and 41 HSPs in resistant genotypes. Expression of HSP to different nematodes is genotype-specific, as studied by Lopes–Caitar et al., (2013) [119] in different soybean genotypes. Six HSP20 showed differential expression to *Meloidogyne javenica* infection. HSP22, -17.9a, -17.9b and -17.4 showed up-regulation in nematode-susceptible genotypes, while HSP17.6 and -22.4 showed a differential expression in both the susceptible and resistant genotypes. Fuganti et al., (2010) [120] identified gene *Gm13G176000,* which had a differential expression in soybean nematode-resistant and susceptible genotypes, while screening soybean resistance to nematode *Meloidogyne javanica* using a microsatellite marker. This later revealed that this gene corresponds to *GmHSP17.6,* which was induced highly in nematode infection. Studies also showed that some HSPs work in correlation with RghI protein and other antioxidant-related enzymes to develop resistance against nematodes. Kandoth et al., (2011) [121], while working with the Soybean cyst nematode (SCN) (*Heterodera spp*.), found RghI was differentially expressed, which induced the HSP20 accumulation in both the resistant and sensitive genotypes. Position of *cis*-acting elements in the promoter region of HSPs could be activated in biotic stresses, depending on the distance from the site of transcription. *Meloidogyne incognita* infection in the sunflower, HSP17.7, in transgenic tobacco showed that HSE were activated within 83 base pairs (bp) and HSP17.7 were up-regulated beyond 83 bp [122]. This trend was further confirmed by Barcala et al., (2008) [123] in HSP17.6 and HSP18.6, where they showed transcription within 108 and 49 bps, respectively. This also confirmed that, not only the position of HSE, but also other transcription factors like TATA Box and CAAT Box also interacted and influenced the expression of HSP20 in biotic stresses. HSP90 knockdown in tomatoes showed resistance to tomato root knot nematodes through SGT1 and Mi-1-mediated resistance [124]. HSP90, on the other hand, promoted the nematode infection. Lourenço et al., (2015) [125] silenced HSP90 and iso-citrate lyase (ICL) in tobacco, which resulted in 46–77% reduction in eggs of the root knot nematode (RKN) *Meloidogyne incognita,* as compared to the wild type which showed nematodes can reproduce only in the presence of HSP90 and ICL.

### 5.2. Abiotic Stress Tolerance

Abiotic stresses are extreme environmental conditions like extreme temperatures, water deficit, and ion imbalance due to heavy metals and salinity, which pose a serious threat to plants survival, yield and quality. Global warming and climate change, due to industrialization, has further worsened the situation. Greenhouse gases, particularly the concentration of CO_2_, is increasing constantly in the atmosphere, which is estimated to reach 520 ppm from 410 by the year 2100 [36]. Twenty per cent of the world cultivated land and almost 50% of irrigated land is affected by salinity, and yield loss up to 50% due to drought is projected by the year 2050 [128]. The world population by the year 2050 is approximated to be 9 billion [129], so, in such a situation, biotic and abiotic stress-tolerant cultivars need to be developed using transgenic and omic techniques. The role of HSPs has been studied by various scientists under different abiotic stresses (Table 3).

#### 5.2.1. Temperature Stress

Extreme temperatures are the potential and important environmental stresses that affect plant survival in many ways. Temperatures stresses are discussed in the following sub-sections.

##### High-Temperature Stress

Accompanying climate change, high temperatures have threatened all organisms, but plants, being directly exposed and unable to change their position, are the most affected [130]. High temperature affects the macro-molecules, like proteins, by misfolding, aggregation and denatured enzymes. It also affects membrane fluidity and its disruption results in the accumulation of ROS and the development of secondary stress [131,132]. Multilevel interactions exist between HSPs and ROS. Plants are wise enough to use ROS as a signal molecule to produce HSPs and other stress-related proteins [85,133].

Several studies indicated that many high molecular weight HSPs showed response under high-temperature stress, such as HSP118, -114, -110, -108, -104, -103, -101, -100 and -97, respectively, [72,134]. Among the HSP100 class, significant HTR was shown by HSP101 [65] and also was involved in thermo-tolerance in *Arabidopsis* [135]. It was confirmed further in maize that HSP101 was involved in thermo-tolerance [70]. Merret et al., (2017) and Mcloughlin et al., (2016) [136,137] studied this trend a step further in *Arabidopsis*; they confirmed the role of HSP101 in thermotolerance, but also established that this played a role in recovery after heat shock. Besides the HSP101 role as a chaperone, HSP100 also was involved in development [138]. Low molecular weight HSPs i.e., -18.1 and -17.9, accumulated in the pea while it was treated for four hours at 42 °C. The response and expression of the HSPs also were development stage and different tissue specific. Maize subjected to 40 °C, HSPs (-101, -70 and -17.6) were induced. Above 36 °C, fertilization was reduced, although HSPs were induced in female reproductive parts but, when studied, mature pollens were more sensitive to heat stress [139]. To contrast, HSP70s were expressed more in tomato pollens [140]. Arabidopsis HSP70 expressed more in mitochondria under high temperature stress [141]. Chloroplast HSP70.1, 70.2 and mitochondrial HSP22 also were involved in seed development aside from its role as a chaperone [142,143]. HSP90 also showed increased expression under heat stress situations. HSP90.1 has been reported in rice and *Arabidopsis* [144,145] and all classes of HSP90 (A, B, and C) in soybeans [146]. Under normal conditions, HSP90 negatively regulated HSF and kept the regulation of all HSP checked [147]. HSP70s and HSP60s chaperonin families are the most studied of the chaperones under heat stress, which maintained protein proper folding using ATPs [38]. Cytosolic HSP70 was involved in heat stress tolerance in *Arabidopsis* [148]. HSP70s have been studied under high temperature stress in a variety of plant crops; such as witch-grass and alfalfa [149,150]; vegetables like pepper, tomato, cabbage, potato; ornamental plants like chrysanthemum [151,152,153,154,155,156,157]; grain such as wheat [158]; and tea [159]. Xu et al., (2010) [160] observed the expression pattern of chloroplast HSP60, not only in normal conditions but, also, under high-temperature and drought situations. It was responsible for Rubisco assembly, protection and also chloroplast development. Low molecular weight HSPs such as the HSP10, HSP20 and HSP40 families were up-regulated under high-temperature stress situations in various plant crops [5,152,161,162,163,164,165]. Some small HSPs were also genotype-specific and were up-regulated in resistant cultivars, such as foxtail millet, while some small HSPs were down-regulated in sensitive genotypes [53]. Some co-chaperones were involved in thermo-tolerance as HSP40. Correlation of small HSPs with HSP100, HSP70 and HSP60 suggested their role as holders in disaggregation and protein folding [157].

##### Low-Temperature Stress

Cold stress affects plant enzymes, membrane plasticity, changes physiology and metabolism, sometimes causes water starvation and desiccation which creates a stress condition for the plant that adversely affects plant growth, development and yield. Low temperature also is associated with protein disfunction and denaturing, which induce the accumulation of HSPs [166,167,168]. Many HSPs responded to cold stress and were up-regulated in *Arabidopsis*, tobacco, maize, rapeseed, chicory, poplar, wheat and barley [167,169,170,171,172,173,174,175,176]. Under low-temperature stress situations, HSPs were induced and translocated into various cell organelles to protect them from cold stress [169]. Bae et al., (2003) [169] investigated this in *Arabidopsis* treated with cold stress at 40 °C for 6 h. *HSP70* were up-regulated and their traffic from the cytoplasm to the nucleus was observed. A similar event was observed in the pea mitochondria when treated at 4 °C for 36 h [177]. Some HSPs accumulated tissue specifically upon low-temperature exposure, as in poplar where HSPs were accumulated in leaves [178]. Regarding rice, low-temperature stress and a gradual decrease in the temperature from 15 °C to 0 °C, with an interval of 5 °C, up-regulated *HSP95* and -75 and HSP70 accumulated in the chloroplast, as this was the vulnerable part of the plant to low temperature [179,180]. Some of the HSPs, like HSP90 in wheat and HSP60 and HSP21 in sunflowers down-regulated to cold stress [175,181]. A similar trend also was reported by Hlavackova et al., (2013) and Rinalducci et al., (2011) [167,182] where Rubisco stability was associated with down-regulation of HSP60 and -21 in winter wheat.

#### 5.2.2. Drought Stress

Drought is the crucial and threatening abiotic factor that limits the productivity of many crops in the current changing climatic conditions. This stress, in combination with other abiotic stresses such as high light and temperature stress, negatively affects plant morphological, physiological and molecular characteristics, which leads to lowered photosynthesis, hormonal imbalance, mineral nutrient starvation and an ultimate oxidative stress [230]. Removal of water disrupts the normal structure of the lipid bilayer plasma membrane. This results in the displacement of membrane proteins, denaturation of membrane-based enzymes and, as a result, membrane permeability, physiology and metabolism are adversely affected [231,232]. Dehydration stress also affects the quantity and quality of normal plant proteins and, as a result, stress related proteins including HSPs are induced. HSP70 was up-regulated in drought stress in the seedling of upland rice [207]. Similarly, transgenic *Arabidopsis* and sugarcane also showed HSP up-regulation and demonstrated drought tolerance [204,211]. The expression pattern of *HSPs* is also genotype-specific. Burke et al., (1985) [212] studied combined drought and heat stress in irrigated and non-irrigated cotton, where more HSPs accumulated in non-irrigated cotton. Maize heat tolerant and sensitive cultivars were studied under high temperature and dehydration situations, where HSP accumulation was more in drought stress conditions [233]. The same was demonstrated by Benesova et al., (2012) [39], where HSP70 and HSP26 were induced in drought-stressed maize. A study on chickpea HSP70 reported that HSPs were first down-regulated in the early stage of growth in drought-tolerant cultivars. To contrast, HSPs were abundant in drought-sensitive cultivars, which indicated that HSPs responded to drought not only in the specific genotypes but, also, during the developmental stage. Similarly, small HSPs expressed highly in drought-tolerant cultivars as compared to those that were sensitive in chickpea [210]. The same trend also was observed in poplar and Kentucky bluegrass [160,213]. HSP17.7 showed drought tolerance in transgenic rice, and other HSPs also were involved in the acclimation of bryophytes to drought stress [209,234]. Proteomics studies revealed that nuclear and HSPs in the extracellular matrix were both up-regulated to drought stress [235,236,237,238].

#### 5.2.3. Salinity Stress

Increased level of salt in cultivable land is also a limiting factor for agricultural production. Twenty percent of cultivable land, and nearly half of the world irrigated land, is affected by salinity [128]. Studies show that many HSPs are induced and up-regulated in saline stress situations like *HSP70* in rice seedlings [197], wheat [195], and poplar *HSP70-9,-12* and -*33* [203]. Furthermore, *HSP40* in rice [165] and poplar, *HSP100*-*21* and *-75*), *HSP90*-*9* and *-12*), *HSP60*-*31, -33, -38* and *-49*), *HSP40*-*113* and -*117*, and *HSP21* were also up-regulated under salt stress [203]. Seen in wheat hybrid Jinan 177 and its salt-resistant hybrid, protein profiling showed HSPs and chaperones were induced highly under salt stress [196]. HSPs were studied in relation to programmed cell death (PCD) in a rice root higher salt situation, where mitochondrial HSP70 were the up-regulated proteins that possibly were involved in PCD regulation [198]. Soybean proteomic studies showed a differential *HSPs* expression of *HSP90,* chloroplast *HSP70, HSP60* and *HSP20* under salt stress [201]. Different HSPs in *Arabidopsis* like HSP 90 [146,239], HSP100, Clp (B1, B2), Clp (D1, D2) and small HSPs in rice [199,240] showed tolerance to high salinity stress. The role of HSPs in response to salinity stress is also genotype-specific, as recorded in soybean, where HSPs were induced more in salt resistant cultivars [202].

#### 5.2.4. Light Stress

Plants, being autotrophs, require light for photosynthesis. Excess light damages the photosynthetic apparatus and plants undergo a phenomenon known as photorespiration. During this process, toxic chemicals rather than sugars, along with ROS, are produced. These toxic chemicals in chloroplast could damage the photosystem II permanently by excessive absorption of light [241]. Rossel et al., (2002) [215] reported many HSPs were up-regulated upon high light stress in *Arabidopsis*. A similar over-accumulation of nuclear HSP70 was observed in *Chlamydomonas* [216]. The thylakoid proteome analysis of *Arabidopsis* was studied with respect to high light saturation involving isoforms of chloroplast HSP70 along with the accumulation of other osmolytes like anthocyanins and ascorbates [214]. Seen in the marine ecosystem where low light created a stress, HSP70, ClpB1, Sti, and HSP60 were up-regulated [242]. Under HLS, small HSP23 was seen to be involved in the post-transcriptional regulation in *Chenopodium rubrum* cell suspension [69].

#### 5.2.5. Chemical Pollutant Stress

Plant productivity is restricted by chemical pollutants in the soil media, such as heavy metals. These pollutants affect plant growth either by displacement of essential cations from specific binding sites or generation of oxidative stress by the generation of ROS. One direct way of disruption was, upon uptake into the cell, a direct reaction took place with proteins due to an affinity for Thionyl^-^, Histidyl^-^ and Carboxyl^-^ groups [243,244].

Many HSPs were induced by heavy metal stress. HSP70s were differentially expressed and accumulated in the roots of tomatoes [217]. Similarly, the HSP70 sub-family, DnaK (Bip), was up-regulated in rice seedlings [218]. *Arabidopsis* exposure to cadmium stress induced many HSPs [222]. Similarly, increased expression was reported for HSP80 and HSP17.9 in rice [219], HSP 90s in *Lotus corniculatus* [245], HSP17.7 in carrots [226] and HSP26 in soybeans [220] under cadmium, lead and arsenic stresses. Using a comparative proteomic analysis of poplar under cadmium stress, a differential expression pattern of HSP was noted. Similarly, in soybeans, two-folds higher accumulation of HSP was recorded in Cd-accumulating genotypes, while there was less HSP70 expression in lower Cd-accumulating varieties, which showed that HSP expression was also genotype-specific [246]. When flax was cultured on heavy metal treated media, many heavy metal binding proteins, including HSP70 accumulation, were enhanced, while HSP83 showed a down-regulation [168]. HSP90.3 enhanced Cadmium stress tolerance by lowering germination potential in *Arabidopsis*, mediating the antioxidant enzymes [247].

#### 5.2.6. Flooding Stress

Waterlogging/flooding is also an environmental limiting factor that hinders plant growth and development. A gradual decrease of redox potential and oxygen in the soil are the ill effects of flooding [248]. Studies show that HSPs are involved in plant resistance against flooding stress by up-regulation and higher gene expression, which is organ-specific. As noticed by Chen et al., (2014) [228] in the soybean plasma membrane where HSP70 accumulated more than 10 folds, this occurrence was more in cotyledon than the roots of the soybean. Discussed in another proteomic study by the same group of researchers, HSP60 was differentially regulated in soybeans [229]. To contrast, HSPs were induced in flooding stress, but were not mandatory for resistance in flooding stress and were genotype specific. Regarding resistant and susceptible cultivars of rice to anoxia and hypoxia conditions, HSPs were more up-regulated in the sensitive cultivars than resistant genotypes [230]. Proteomics study of flooding stress in relation to PCD in maize revealed that HSP70s were more up-regulated [231]. The same pattern of flooding tolerance was studied in rice protoplast where ectopic mtHSP70 expression protected H_2_O_2_ induced PCD [230]. Similarly, *Arabidopsis* showed that anoxia tolerance was enhanced via HSFA2-mediated production of HSP70 and HSP101 [224], which indicated the involvement and multifarious role of HSPs in flooding situations.

#### 5.2.7. Oxidative/Combined Stress

**Figure 3:**
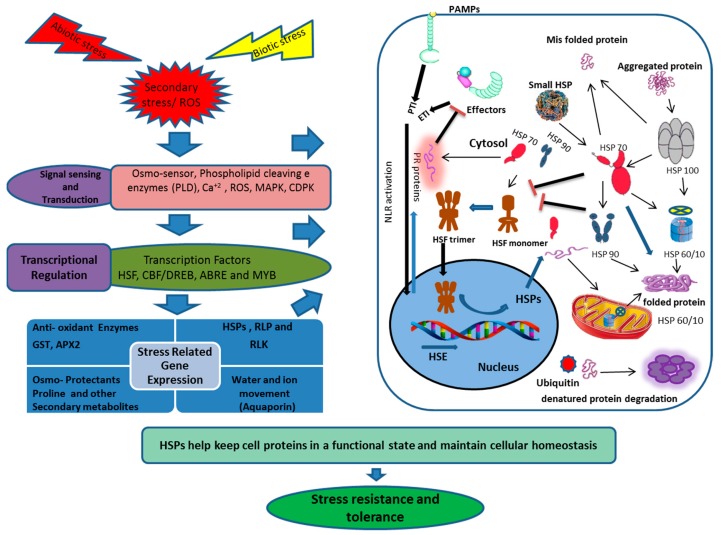
Schematic presentation of the HSP transcriptional regulation, transport and disposal, under biotic and abiotic stresses. The diagram integrates both positive (Arrows) and negative (Bars) regulatory mechanisms. Biotic and abiotic stresses provoke the HSFs through calcium accumulation, recognition of invading pathogen effector proteins, ROS or misfolding and aggregation of cell proteins, which results in activation of HSP and other stress responsive proteins. ROS (Reactive oxygen species), PLD (Phospholipase D), MAPK (Mitogen-activated protein kinase), CDPK (Calcium-dependent protein kinase), HSF (Heat shock factor), CBF (C-repeat binding factor), DREB (Dehydration response element binding protein), ABRE (Abscisic acid-responsive element), MYB (Myeloblastosis), HSP (Heat shock protein), RLKs (Receptor-like kinases), RLP (Receptor-like proteins), PAMP (Pathogenesis-associated molecular pattern), PTI (Pattern-triggered immunity), ETI (Effector-triggered immunity) NLR (Node-like receptor protein), GST (Glutathione-s-transferase), APX2(Ascorbate peroxidase 2).

Since plants are exposed to many stresses simultaneously, such as light, this creates high temperature stress that leads to dehydration. Such situations lead to oxidative or secondary stress and plants have to adjust their signaling pathways and metabolisms to ensure their growth and development [132,197,249,250]. Oxidative stress generates ROS which, in high concentrations, are harmful to cellular structures. HSPs respond to multiple stress situations and enable the plants to cope with the challenging environment. HSP70 expression was higher in tobacco to heat stress but was even higher to the combined stress of heat and drought [251]. Ectopic expression of genes from soybeans in *Arabidopsis GmHSP90* showed tolerance to heat, salinity and osmotic stresses, although response in salinity was not as high as to combined stresses [146]. A similar pattern was observed with small HSPs in rice to multiple stresses [252,253]. Overexpression of *HSP17.6* in *Arabidopsis* enhanced tolerance to salinity combined with dehydration, but no response was noted to high temperature stress only [254]. Single or combined stresses led to the production of ROS and oxidative stress which, if not checked timely, are very detrimental to plants [248] (Figure 3). Under oxidative stress, overexpression of organelle and cytosolic *HSP90* enhanced tolerance in *Arabidopsis*. Similar results were reported by Nishizawa-Yokoi et al., (2010) [255], where *HSP90* regulated HSFA2, which enhanced tolerance to oxidative stress. Queitsch et al., (2000) [135] reported oxidative stress accumulated HSP100/Clp B, ClpC2 and ClpD1 in rice. HSPs protected vital cellular parts under oxidative stress, as demonstrated by Downs et al., (1999) [256], where small HSPs protected the photosystem II from oxidative stress and photo-inhibition. Different organelle HSPs also responded to oxidative stress. mtHSP22 accumulation was enhanced in tomatoes under oxidative stress [257]. Small HSPs responded to oxidative stress, as HSP16.4 and HSP17 accumulated in multiple stress situations in *Arabidopsis* and carrots, respectively [16,223].

## 6. Conclusions and Future Prospects

Plants are exposed to several biotic and abiotic stresses, which not only limit the performance of the plants in term of productivity, but also their quality and storability. Plants have evolved various morphological, anatomical, physiological, phonological and molecular level strategies to deal with stress situations (Figure 1). Plants respond to stress at the molecular level by transcriptional regulation of stress-related proteins, including HSPs. HSPs are classified into different classes, based on approximate molecular weight, as HSP100, -90, -70, -60 and small HSPs. HSPs prevent protein aggregation and maintain a non-native protein’s functional conformation and cell homeostasis under stress situations. The majority of HSPs are up-regulated under various biotic and abiotic stresses, while a few are down-regulated (Table 1). Moreover, HSPs as chaperones also play a role in membrane stability [228], using ROS as a signal molecule and scavenging by positively regulating the antioxidant enzymes [85,115,133], along with plant growth and development under normal conditions [43,46,258,259]. Small HSP (Ubiquitin) also is responsible for denatured protein degradation and disposal. Stress response is a complex process; HSPs play a pivotal role in stress response and can be employed in transgenic plant development. Studies on HSPs are limited to model plants under controlled laboratory conditions; many studies in other plant crops are limited to gene expression due to lack of suitable mutants. HSPs have a wide range of members, and every member plays a significant role across different networks. The response of HSP is genotype and tissue specific. so the signal perception and cascade need to be explored fully in control and stress situations. The response of HSPs to biotic stresses is evident, for example, but signal sensing and regulation are not yet fully elucidated.

Someday, a combination of advanced technologies, such as microarray and omics techniques, at different developmental stages, genotypes and under different biotic and abiotic stresses, alone or in combination, will lead us to better understand the role of HSPs and the associated signaling pathways. More studies are needed, with a focus on different important crop plants under natural field conditions, with respect to the HSP network. Additionally, future research should be extended to explore other regulatory mechanisms such as alternative splicing, miRNAs and their interaction and cross talk with the complex HSFs, HSPs, phyto-hormones and protective enzymes on plant growth, development and metabolism, under normal and stress conditions. This will provide avenues for the development of stress-resistant and tolerant crops through biotechnological approaches and molecular breeding.

## Figures and Tables

**Table 1:** Nomenclature of HSPs in prokaryotes and eukaryotes.

Bacteria (*Escherichia coli*)	Eukaryotic Cell
Caseinolytic protease (Clp B)	HSP100
High Temperature Protein (Htp G)	HSP90
Dna k	HSP70
GroEL	HSP60
Dna J	HSP40
Ibp A	HSP20
GroES	HSP10

**Table 2:** Summary of the role of plant HSP under biotic stresses.

Biotic Factor	Plant	Pathogen	Disease Caused	HSP Response	Expression Pattern	Reference
Viruses	*Arabidopsis thaliana*	TVCTV, ORTV, PVX, CMV, and TuMV	Penstemon disease, red spots on leaves, deformed leaves and stunted growth	HSP17.6 HSP17.4	Up/down	[110]
	*Solanum lycopersicum*	TYLCV	Stunted and bushy growth and excessive branches, abnormal leaf shapes, curled inward or upward, and flower and fruit drops	HSP70 HSP90	Up/down	[109,110,111]
	*Nicotiana benthamina*	CNV, RCNMV, and TMV	Mosaic and discoloration on leaves of a wide-range of the host plant	HSP70 HSP90	Up	[108,112,113]
	*Solanum tuberosum*	SYNV and INSV	Yellow and black rings, spots and lesions on leaves, and leads to plant death	HSP18 HSP20	Up	[114]
	*Oryza sativa*	RSV and RBSDV	Dark green rigid leaves, white- and sometimes black-streaked strips along the leaves, veins and stem	HSP20 HSP70	Up	[7,115,116]
	*Solanum tuberosum*	PVY	Potato tuber necrotic rings, spots, disease and decay	HSP	Up	[117]
Bacteria	*Nicotiana benthamina*	*Ralstonia solanacearum*	Wide- range of the host, it enters the xylem of a plant and causes wilting	HSP17	Up	[103]
		*Ralstonia solani*	Bacterial wilt by blockade of conducting vessels	HSP90	Up	[107]
	*Citrus spp*	*Xanthomonas axonopodis pv. citri*	This bacterium causes the citrus canker and spots on leaves and blemishes on fruits.	Hsp15.5	Up	[104]
	*Capsicum annuum*	*Xanthomonas campestris pv. Vesicatoria*	Causes leaf and fruit spots on peppers and tomatoes.	Hsp16 HSP20	Up	
	*Arabidopsis thaliana*	*Pseudomonas syringae*	Round to irregular brown spots. These spots enlarge and blight the whole leaves.	HSP17 HSP21 HSP23	Down	[105,106]
Fungi	*Oryza sativa*	*Magnaporthe grisea*	Causes destructive disease of rice, rice blast, rice seedling blight and pitting disease.	HSP16 HSP17.4 HSP18	Up	[92]
	*Solanum lycopersicum*	*Fusarium oxysporum*	Wilting, characterized by clearing of veins, marginal necrosis, yellowing of lower leaves, adventitious roots and ultimate death of tomato plants.	HSP20	Up	[93]
		*Rhizopus nigricans Ehrenb.*	Rhizopus soft rot first appears as water-soaked areas, which then become sunken and gray mold and dusky black spores grow on the fruit surface of tomatoes.	HSP17.6	Up	[94]
		*Oidium neolycopersici*	Fungus that causes white powdery lesions and powdery mildew on tomatoes	HSP72 HSP75	Up	[99]
	*Arabidopsis thaliana*	Rhizoctonia solani	Fungus which causes collar rot, root rot and damping off	HSP17.4 HSP17.6	Up	[86,87]
	*Malus domestica*	*Venturia inaequalis*	Fungus causes scab disease on apples and pears	HSP21	Down	[98]
	*Solanum tuberosum*	*Phytophthora infestans*	Fungus causes late blight of potatoes	HSP17.8 HSP70	Up Up	[100] [101]
	*Hordeum vulgare*	*Blumeria graminis f. sp. hordei*	Fungus causes powdery mildew on grasses and cereals like barley.	Hsp16.9 Hsp17.5.	Up	[118]
Nematodes	*Gossypium hirsutum*	*Roylenchulus reniformis*	Stunted growth, root necrosis and the plant shows symptoms similar to nutrient and water deficiency	54 HSP	Up	[7]
	*Glycine max*	*Meloidogyne javanica*	Root knot on tropical crops, it is causes irregular galls and swollen roots	HSP22.4 HSP17.9 HSP17.9 HSP22.4	Up	[119,120]
		*Heterodera glycines*	Forms cysts on the roots of soybeans. It causes chlorosis of leaves and stem and root necrosis	HSP20	Up	[121]
	*Helianthus annuus*	*Meloidogyne incognita*	Irregular galls on the root of sunflowers	HSP17.6 HSP17.7 HSP18.6	Up	[122,123]
	*Solanum lycopersicum*	*Meloidogyne spp.*	Knots and galls on the roots of tomatoes, swollen roots and dwarf stem	HSP90	Up	[124]
	*Nicotiana benthamina*	*Meloidogyne incognita*	Root knot and galls on tobacco roots and causes wilting of leaves	HSP90	Up	[125]

Turnip vein clearing virus (TVCTV), Oilseed rape virus (ORTV), Potato virus X (PVX), Cucumber mosaic virus (CMV), Turnip mosaic virus (TuMV), Tomato yellow leaf curl virus (TYLCV), Cucumber Necrosis Virus (CNV), Red clover necrotic mosaic virus (RCNMV), Tobacco mosaic virus (TMV), Sonchus yellow net virus (SYNV), Impatiens necrotic spot virus (INSV), Rice stripe virus (RSV), Rice black Streaked dwarf Virus (RBSDV), Potato virus Y (PVY).

**Table 3:** Summary of studies of plant HSP and abiotic stresses.

Abiotic Factors	Plant	Type of HSP	Expression Pattern	Technique Used	Reference
High temperature stress	Wheat	HSP70	up	qRT-PCR	[158]
		HSP26	up	qRT-PCR	[183]
	Rice	HSP100	up	WB	[134]
		HSP90	up	q-PCR	[145,146,184]
	Maize	HSP101	up	SDS-PAGE	[63,65]
		HSP70, HSP17.6	up	SDS-PAGE	[139]
	*Arabidopsis*	HSP101	up	qRT-PCR	[127,128,129,130]
		HSP100	up	SDS-PAGE	[65]
		HSP90	up	qRT-PCR, WB	[147,185]
		HSP70	up	qRT-PCR	[186]
		HSP60 HSP70	up	qRT-PCR	[148]
	Potato	HSP70	up	qRT-PCR	[156]
	Tomato	HSP70	up	SDS-PAGE, WB	[140]
		HSP20	up/down	qRT-PCR	[187]
	Pea	HSP17.9 HSP18.1	up	qRT-PCR	[188]
		HSP70	up	qRT-PCR	[154]
	Pepper	HSP70	up/down	qRT-PCR	[151]
		HSP70	up	qRT-PCR	[153]
		HSP60	up	qRT-PCR	[189]
		HSP20	up/down	qRT-PCR	[78]
		HSP16.4	up	qRT-PCR	[152]
	Soybean	HSP90	up	qRT-PCR	[146]
	Cabbage	HSP70	up	qRT-PCR	[155]
	Tea	All HSPs	up	qRT-PCR	[159]
	Witch grass	HSP70	up	MA, qRT-PCR	[149]
	Alfalfa	HSP70	up	qRT-PCR	[150]
	Foxtail millet	HSP20	down	qRT-PCR	[53]
	Chrysanthemum	HSP70	up	MS, qRT-PCR	[157]
Low temperature stress	*Arabidopsis*	HSP70	up	MS, qRT-PCR	[169]
	Tobacco	HSP70	up	MS, qRT-PCR	[161,165]
	Maize	HSP70	up	MA, qRT-PCR	[171]
	Wheat	HSP70	up	MS	[190]
		HSP90	up	MS	[175]
		HSP60 HSP21	down	MS, qRT-PCR	[167]
	Rice	HSP75 HSP95	up	MS, qRT-PCR	[180]
		HSP90	up	MS	[191]
	Barley	HSP70	up	MS	[167]
	Chicory	All HSPs	up	MS	[173]
	Rape seed	HSP90	up	qRT-PCR	[172]
	Poplar	HSP70 HSP90	up	MS	[178]
	Pea	HSP70	up	MS	[177]
	Sunflower	HSP60 HSP21	down	MS, qRT-PCR	[181]
	Tomato	HSP110 HSP70	up	qRT-PCR	[192]
	Plum	HSP20	up	qRT-PCR	[193]
	Grape	HSP18 HSP22	up	qRT-PCR	[194]
Salinity stress	Wheat	HSP70	up	MS	[195]
		All HSPs	up		[196]
	Rice	HSP70	up	qRT-PCR	[197,198]
		HSP40	up	qRT-PCR	[165]
		ClpD1	up	qRT-PCR	[199]
	*Arabidopsis*	HSP90.2 HSP90.5 HSP90.7	up	qRT-PCR	[200] [146]
	Rose	17.8	up	qRT-PCR	[16]
	Soybean	HSP90 HSP70 HSP60 HSP20	up/down	MS	[201] [202]
	Poplar	HSP100 HSP90 HSP70 HSP60 HSP40 HSP20	up	qRT-PCR	[203]
Drought stress	*Arabidopsis*	HSP70	up	qRT-PCR	[204]
	Tobacco	HSP70 BiPs	up	qRT-PCR	[205] [176]
	Barley	HSP17.5	up	qRT-PCR	[206]
	Rice	HSP70	up	MA, MS, qRT-PCR	[207]
		HSP101	up	MS	[208]
		HSP17.7	up	qRT-PCR	[209]
	Maize	HSP70 HSP26	up	MS	[39]
	Pepper	HSP16.4	up	qRT-PCR	[152]
	Chickpea	HSP70	up	MS, qRT-PCR	[210]
	Sugarcane	HSP70	up	qRT-PCR	[211]
	Cotton	All HSPs	up	qRT-PCR, WB	[212]
	Kentucky grass	HSP70	up	MS	[160]
	Poplar	HSP70	up	MS	[213]
Light stress	*Arabidopsis*	HSP70	up	qRT-PCR	[214,215]
	Goose foot plant	HSP23	up	SDS-PAGE	[69]
	Chlamydomonas	HSP70	up	MA	[216]
Heavy metal stress	Tomato	HSP70	up	MS, SDS-PAGE	[217]
	Rice	HSP70 BiPs	up	MS, SDS-PAGE	[218]
		HSP80 HSP17.9	up	MA	[219]
	Poplar	HSP70	up	qRT-PCR	[212]
	Soybean	HSP26	up	qRT-PCR	[220]
	Carrot	HSP17.7	up	qRT-PCR	[221]
	Flaxseed	HSP70	up	MA, MS	[168]
		HSP80	down		
	*Arabidopsis*	HSP70	up	qRT-PCR, NB, MS	[222]
	Bird foot trefoil	HSP90	up	qRT-PCR	[223]
Flooding stress	*Arabidopsis*	HSP101 HSP70	up	qRT-PCR	[224]
	Tomato	HSP23.6	up	qRT-PCR	[225]
	Rice	HSP70	up	qRT-PCR	[226]
	Maize	HSP70	up	qRT-PCR, WB	[227]
	Soybean	HSP70	up	qRT-PCR, SDS-PAGE	[228]
		HSP60	up	MS	[229]

Quantitative real-time polymerase chain reaction (qRT-PCR), Mass spectrometry (MS), Microarray (MA), Western blotting (WB), Northern blotting (NB), Sodium dodecyl sulfate–polyacrylamide gel electrophoresis (SDS-PAGE).
